# The long and winding road: From mouse linkage studies to a novel human therapeutic pathway in type 1 diabetes

**DOI:** 10.3389/fimmu.2022.918837

**Published:** 2022-07-22

**Authors:** Manuel Rojas, Luke S. Heuer, Weici Zhang, Yi-Guang Chen, William M. Ridgway

**Affiliations:** ^1^ Division of Rheumatology, Allergy and Clinical Immunology, University of California, Davis, Davis, CA, United States; ^2^ School of Medicine and Health Sciences, Doctoral Program in Biological and Biomedical Sciences, Universidad del Rosario, Bogota, Colombia; ^3^ The Max McGee Research Center for Juvenile Diabetes, Children’s Research Institute of Children’s Wisconsin, Milwaukee, WI, United States; ^4^ Division of Endocrinology, Department of Pediatrics, The Medical College of Wisconsin, Milwaukee, WI, United States

**Keywords:** NOD, T1D (type 1 diabetes), t cell, treg cells, CD137, CD137L

## Abstract

Autoimmunity involves a loss of immune tolerance to self-proteins due to a combination of genetic susceptibility and environmental provocation, which generates autoreactive T and B cells. Genetic susceptibility affects lymphocyte autoreactivity at the level of central tolerance (e.g., defective, or incomplete MHC-mediated negative selection of self-reactive T cells) and peripheral tolerance (e.g., failure of mechanisms to control circulating self-reactive T cells). T regulatory cell (Treg) mediated suppression is essential for controlling peripheral autoreactive T cells. Understanding the genetic control of Treg development and function and Treg interaction with T effector and other immune cells is thus a key goal of autoimmunity research. Herein, we will review immunogenetic control of tolerance in one of the classic models of autoimmunity, the non-obese diabetic (NOD) mouse model of autoimmune Type 1 diabetes (T1D). We review the long (and still evolving) elucidation of how one susceptibility gene, *Cd137*, (identified originally *via* linkage studies) affects both the immune response and its regulation in a highly complex fashion. The CD137 (present in both membrane and soluble forms) and the CD137 ligand (CD137L) both signal into a variety of immune cells (bi-directional signaling). The overall outcome of these multitudinous effects (either tolerance or autoimmunity) depends upon the balance between the regulatory signals (predominantly mediated by soluble CD137 *via* the CD137L pathway) and the effector signals (mediated by both membrane-bound CD137 and CD137L). This immune balance/homeostasis can be decisively affected by genetic (susceptibility vs. resistant alleles) and environmental factors (stimulation of soluble CD137 production). The discovery of the homeostatic immune effect of soluble CD137 on the CD137-CD137L system makes it a promising candidate for immunotherapy to restore tolerance in autoimmune diseases.

## Introduction

Autoimmune diseases (ADs) are a chronic and clinically heterogeneous group of diseases affecting up to 5% of the world population (1, 2), and their incidence is rising (3). Different ADs share risk factors (e.g., environmental and genetic) and immunological mechanisms (4). A single autoimmune disease may manifest with autoantibodies of diverse organ specificities (i.e., latent polyautoimmunity) (5–7). Polymorphisms in *HLA-DRB1, HLA-DQB1, CD226, PTPN22, STAT4, GPR103, TNFAIP3*, and *LRP1/STAT6* are associated with multiple ADs (8, 9), including systemic and organ-specific ADs (10). Therefore, the study of autoimmunity is complex and requires the analysis of multiple genes with diverse immunological effects.

A commonality among ADs is the failure to control peripheral autoreactive T cells, and most ADs exhibit dysfunctional T regulatory cells (Tregs) (11). This T cell population constitutively and highly expresses CD25 (IL-2 receptor α chain) (12), and more specifically, Tregs express the transcription factor Forkhead box P3 (FOXP3) (13–15). The clinical relevance of FOXP3 was demonstrated in patients with the immune dysregulation polyendocrinopathy enteropathy X-linked (IPEX) syndrome (16). More than 70 mutations in *FOXP3* have been described in these patients (17), and they exhibit a high frequency of polyautoimmunity, such as autoimmune thyroid disease, autoimmune cytopenia, or type 1 diabetes (T1D) (18). Polymorphisms in other genes implicated in Treg function, such as *IL2RA* and *CTLA4*, have also been associated with the development of endocrinological and rheumatic ADs (19, 20). This evidence highlights the crucial role of Tregs in the disrupted immune homeostasis characteristic of autoimmunity.

The current management of ADs is centered on immunosuppression. Multiple non-specific immune-suppressive therapies are used to ameliorate autoreactivity/tissue damage (i.e., methotrexate, leflunomide). More recently, antibody-based therapies target specific molecules or cells involved in the immune response (i.e., anti-CD20 for depleting B cells) (21). However, these approaches have a major undesired effect: increased susceptibility to infections. Recently, new therapeutics focusing on Tregs have emerged. For example, administration of IL-2 in patients with systemic lupus erythematosus (SLE) ameliorated disease *via* the expansion of Tregs without an increased risk of infection, and low dose IL-2 therapy is being investigated in T1D (22–25). Restoring Treg function might treat autoimmunity while reducing the risk of life-threatening adverse effects. However, abnormal Treg function and conversion of Tregs to pathogenic Th17 cells are complications in Treg therapeutics (26–28).Thus, deeper knowledge of Treg biology is needed.

T1D is one of the most common ADs in children, characterized by the autoimmune destruction of insulin-producing β cells (29). T1D incidence is increasing rapidly, implying increasing environmental factors interacting with genetic risk loci (*HLA* and *non-HLA* genes) (29–31). Antigen-presenting cells (APCs) initiate pancreatic inflammation by producing inflammatory cytokines such as TNF-α (32, 33). The presentation of pancreatic antigens by APCs then leads to the activation of autoreactive CD4^+^ and CD8^+^ T cells, which perpetuate insulitis and the destruction of β cells (34, 35). Treg failure to maintain peripheral tolerance of these autoreactive T cells due to Treg dysfunction is critical in the persistence of inflammation and islet destruction (36).

Phase 1 clinical trials on early-onset T1D showed that the administration of autologous expanded CD4^+^CD25^+^CD127^−^ Tregs was associated with a reduced requirement of exogenous insulin and preservation of β-cell function, with this effect persisting for up to 1 year after infusion without severe adverse reactions (37, 38). In a similar study, adult patients showed stable levels of C-peptide and insulin use for up to 2 years (39). However, this Treg strategy would necessitate periodic re-transfusions of Tregs to maintain the immune response, and autologous transplantations of Tregs may be difficult in low-income settings. In addition, these studies are in their infancy (i.e., phases 1 and 2), and the estimated magnitudes of the effect of these approaches were low. Thus, other strategies are needed to boost the peripheral Treg response to restore homeostasis.

## The NOD strain and its implications for T1D research

**Figure 1:**
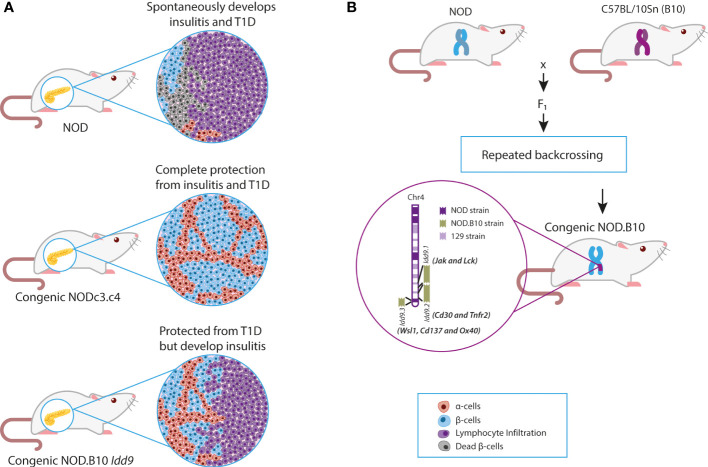
NOD and NOD Congenic mice. (A) Effects of congenic intervals on the clinical and histological phenotypes of NOD mice. (B) Breeding of NOD and B10 mice to produce congenic mice with Idd9 regions. Chr, Chromosome; NOD, Non-obese diabetic; T1D, Type 1 diabetes.

The non-obese diabetic (NOD) mouse, which spontaneously develops autoimmune T1D, has long served as a model to delineate both genetic and immune mechanisms of T1D and its treatment. This model was established in 1980 by Makino et al. (40) and emerged from breeding a mouse strain that spontaneously developed cataracts (i.e., CTS strain) (41). Two groups of mice emerged: males with glucose intolerance but without glucosuria, later known as the non-obese non-diabetic (NON) strain, and females with polyuria, ketoacidosis, and glucosuria, subsequently known as the NOD strain (41). Histological examinations of NOD mice demonstrated lymphocyte infiltration in pancreatic islets (insulitis), as well as a decrease in the number of β-cells and islet size (**Figure 1A** **)** (40).

Typically, 80% of female NOD mice develop insulitis at three weeks and T1D at ~20 weeks (42). The *H2^g7^* MHC haplotype essential for T1D development in NOD mice has the unique I-A allele (I-A^g7^). I-A^g7^ encodes histidine and serine at positions 56 and 57 instead of the two usually conserved proline and aspartic acid residues found in other mouse strains (43). The diabetogenic variants of the human class II HLA-DQβ homolog also have non-aspartic acid substitutions at residue 57 (44). The genetic association of both MHC class I and class II with disease supports the pathogenic role of CD8^+^ and CD4^+^ T cells in the destruction of β-cells in humans and mice (45–48). Multiple autoantigens are targeted by autoreactive T cells (e.g., GAD, insulin, or HSP) (49). However, while the MHC II I-A^g7^ is a major susceptibility allele, it is not sufficient for the development of diabetes, as shown by complete T1D resistance in B10 mice expressing I-A^g7^ molecules (50). In B6 congenic mice expressing I-A^g7^, circulating T cells can react with the same β-cell autoantigens as in NOD mice; however, no autoimmunity results. These B6.G7 congenic mice confirm the importance of non-MHC genes in controlling autoimmunity in NOD genetic background (51, 52).

In addition to CD4^+^ and CD8^+^ T cell autoreactivity, Tregs are involved in suppressing the development of T1D in NOD mice. CD4^+^CD25^+^ Treg cell depletion at critical time points can accelerate T1D progression (53). Ablation of essential proliferative or co-stimulatory signals required for Treg cells, such as IL-2 or CD28, exacerbates T1D (54). NOD Treg quantity and functional capability are reduced, and increasing NOD Treg cell activity can prevent diabetes (55–57). These studies suggested that in addition to the crucial role of T cell autoreactivity, immunological pathways related to Tregs could be genetically determined in the NOD model. Further studies showed shared susceptibility genes affecting Treg function between mice and humans for T1D (e.g., *IL2* and *CTLA-4*) (58–60). Thus, the study of NOD Treg function and control may allow the implementation of novel therapeutics in humans. These considerations highlight the significance of identifying *non-HLA* genes implicated in immune regulatory function, Treg function and development, and T1D pathogenesis (60). Identifying genes in the B6/B10 genetic background that can control autoimmunity has thus been a major goal in this field.

## Immunogenetic studies of NOD and Human T1D and translation to novel therapeutics

Before the advent of whole-genome sequencing, many non-*HLA* genomic regions associated with T1D were discovered by linkage analysis of the NOD genome (61–63). Identified genetic regions were confirmed to play a role in T1D pathogenesis through the construction of congenic mice (64). Congenic mice were constructed by introgression of resistant insulin-dependent diabetes (*Idd*) loci/regions onto the NOD background. Backcrossing of NOD with B6 (C57BL/6J), B10 (C57BL/10Sn), and other T1D resistant strains demonstrated that over 30 murine recessive *Idd* loci were associated with protection from spontaneous diabetes (65) (**Figure 1B**). These studies allowed the classification of *Idd* intervals into two groups: those that confer both insulitis and diabetes resistance and a second group that protects against T1D but has no effect on insulitis (62) (**Figure 1A**). For example, the *Idd3* locus on chromosome 3 was implicated in the protection from insulitis and T1D, whereas the *Idd4* locus on chromosome 11 did not protect from insulitis but prevented T1D (61). It suggested that genes within these regions exhibited differential effects on T1D development (i.e., T cell migration, cytotoxicity, or Treg function). The next step was to identify and confirm candidate genes within the introgressed genetic regions. This confirmation process ultimately has taken decades of work and the development of new technologies (e.g., whole-genome sequencing, CRISPR).

One of the first identified non-*HLA* candidate genes encodes interleukin 2 (IL-2). IL-2 is located in the *Idd3* region and has profound effects on T cell and Treg function, and was thus a good candidate gene for T1D (66). NOD produces an altered IL-2 protein compared to the protective B10 allele, with a shortened tandem repeat sequence encoding a poly-glutamine stretch, plus an extra four amino acid insert, in the N-terminal coding region of IL-2 (62). These immunogenetic studies uncovered evidence of multiple genes with multiplicative effects on the immune response. For example, the *Idd3/Idd5* double congenic mice, comprising the *Il2* and *Ctla4* candidate genes, were completely protected from T1D, whereas when studied alone, only ~20% and ~50% rates of protection were observed, respectively (60, 67, 68).

Genetic studies in the mouse were compared to human T1D genetic studies, and marked similarities were uncovered. The genetic architecture of mouse and human T1D is remarkably similar, with variants affecting multiple immune genes and pathways in common between both species, including *IL-2*, *IL-2* receptor, *CTLA-4*, *IL-10*, the *HLA* region, *PTPN22*, and *IL-7R* (69, 70) For example, single nucleotide polymorphisms (SNPs) in the human homologous *Il2* region were also associated with T1D susceptibility, identifying the IL-2 pathway as potentially shared in the pathogenesis of disease in both species (71). The NOD *Il2* gene variant resulted in decreased production of IL-2, and elegant engineering of *Il2* gene haplodeficency reproduced the NOD effect and resulted in functionally deficient Tregs (71). Low dose IL-2 therapy increased Tregs in mouse models, and this led to human trials of low dose IL-2; however, while this boosted human Treg numbers, it did not affect T1D outcome in initial trials (72). A variety of approaches have tried to optimize immune modulation effects *via* IL-2. IL-2 induced *in vitro* expansion of Tregs is one approach that was effective in NOD mice, tying the IL-2 immunogenetic effects to the enhancement of Treg deficiencies in T1D (73). Large numbers of Tregs are needed for human trials, and *in vitro* expansion may overcome some of the deficiencies of earlier Treg trials (74). Clinical trials in human T1D are ongoing with low-dose IL-2 therapy (75) and Treg therapy (76) which have built upon these earlier results.

CTLA-4 is another critical immune molecule with variants identified in mice and humans. The mouse locus (*Idd5*) was noted to overlie the orthologous human *IDD12* locus (67). T1D susceptibility was subsequently mapped to a non-coding region of human CTLA-4 that resulted in lower levels of the CTLA-4 soluble splice variant; the mouse gene also demonstrated alterations in CLTA4 splicing (77). Human trials targeting CTLA-4 with a soluble form that blocks T cell activation appear promising (78). These therapies may be effective even though the human disease demonstrates remarkably different patterns of insulitis than the mouse, with much less exuberant immune infiltrates (79). The difference in β cell immune infiltration may explain why prevention of diabetes NOD is very easily achieved, whereas, in humans, prevention trials have until recently failed. One successful approach to the prevention of human T1D has been achieved using anti-CD3 antibodies, which preferentially target CD8 effector cells (80). Notably, anti-CD3 antibodies were discovered in NOD mice to reverse established disease (not simply prevent disease), demonstrating the usefulness of therapeutic trials of acute T1D in NOD mice. Overall, these examples illustrate the rich insights and potential therapies resulting from T1D immunogenetic studies. The latest large-scale study identified 78 genetic regions linked to T1D (including 36 novel loci) and confirmed the strong association with immune function and potential for clinical therapeutics (81). Thus much more work can be done to apply immunogenetic studies to novel therapeutic pathways.

Our labs have been investigating immunogenetic control of T1D, initially using NOD and NOD congenic mice, for over 20 years. In the rest of this review, we will detail the lengthy investigation of the immune effects of the *Idd9* genetic region and our studies which demonstrated that *Cd137* is the essential T1D susceptibility gene in this region. These studies have revealed many surprises about the function of an *Idd* gene in T1D immunology and have ultimately led to novel immunotherapy based on the immune function of CD137.

## The role of *Idd9* and its main candidate gene, *Cd137*, in NOD T1D

After identifying the *Idd9* region in linkage studies, the Wicker group constructed congenic mice with the B10 *Idd9* region introgressed onto the NOD background. The B10 *Idd9* region prevented the onset of spontaneous diabetes in NOD mice (less than 5% of female mice developed T1D) (82). However, most mice still developed insulitis caused by T cells expressing CD30, producing high amounts of IL-4 (82) (**Figure 1A**). This confirmed that genes associated with lymphocyte infiltration were outside the *Idd9* interval but suggested that some genes within this region halted autoimmunity.

This hypothesis was validated in double congenic mice comprising B6 (*Idd3, Idd17, Idd10*, and *Idd18*) from chromosome 3 and B10 (*Idd9*) regions (also known as the NOD.c3c4 strain). NOD.c3c4 mice were completely protected from diabetes, and only 10% of mice developed insulitis (82) (**Figure 1A**). This confirmed that spontaneous diabetes is a complex trait in which the epistasis of multiple genes (*HLA* and non-*HLA*) is critical for its development, but it also suggested that the *Idd9* interval contained genes associated with T cell activation and modulation.

The *Idd9* region, a 48 cM interval, was fine-mapped into three intervals (i.e., the *Idd9.1, 9.2*, and *9.3*), with seven candidate genes (i.e., *Jak1, Lck, Cd30, Tnfr2, Cd137, Wsl1*, and *Ox40*) (66). The *Wsl1, Cd137*, and *Ox40* were initially proposed as candidate genes within the *Idd9.3* locus (82). However, B6 *Wsl1* did not exhibit sequence variations compared to NOD, and *Ox40* was subsequently found to be located outside of the *Idd9.3* region and was excluded as a candidate gene (82). Thus, *Cd137* remained the key candidate for T1D protection within the *Idd9.3* locus. Jumping ahead 15 years, it was recently confirmed by using combined congenic mapping and nuclease-based gene targeting that *Cd137* is the susceptibility gene within the *Idd9.3* locus critical for modulation of T1D (82, 83).

**Figure 2:**
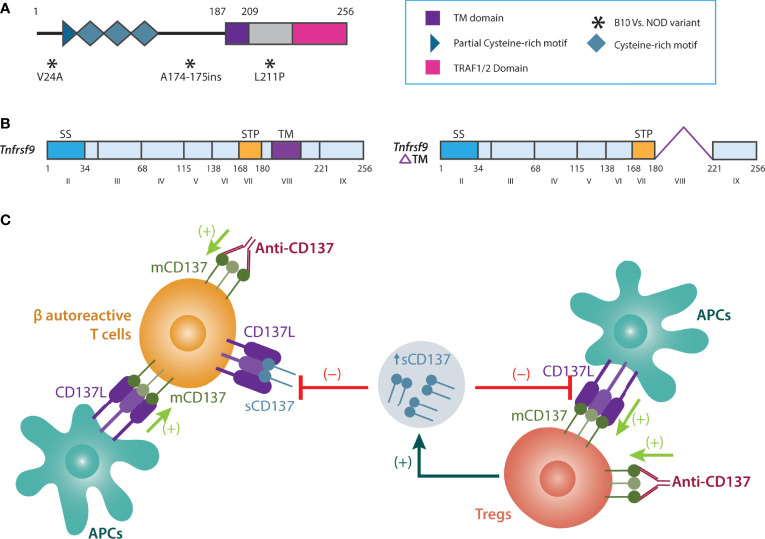
Biology and function of sCD137. **(A)** Non-synonymous SNPs of NOD vs. B6 *Cd137*. **(B)** membrane vs. soluble (alternatively spliced) CD137. **(C)** Tregs produce sCD137 with a dimeric structure. sCD137 induces altered CD137L signaling in APCs and autoreactive T cells (compared to membrane CD137), reducing inflammation and damage in the pancreas. In contrast to sCD137, although anti-CD137 antibodies activate Tregs (a strong immune regulatory effect), they may also increase autoreactive T cell survival and proliferation, thus perpetuating inflammation and autoimmunity. APCs, Antigen-presenting cells; CD137L, CD137 Ligand; mCD137, Membrane-bound CD137; NOD, Non-obese diabetic; sCD137, Soluble CD137; SS, signal sequence; STP, Ser/Thr/Pro-rich; T1D, Type 1 diabetes; TM, Transmembrane domain; Tregs, T regulatory cells.

*Cd137* is located at 1.217-Mb of the *Idd9* locus (i.e., *Idd9.3*) (84), and *Idd9.3* conferred ~40% protection for T1D (82). Analysis of coding variants demonstrated two synonymous SNPs in NOD vs. B10 *Cd137*: a valine to alanine substitution at position 24 and leucine to proline substitution at position 211 (near the transmembrane domain). There is also alanine insertion in NOD between amino acids 174 and 175 (82) (**Figure 2A**). These structural modifications suggested that CD137(4-1BB) could be hypofunctional in NOD mice (82). *Cd137 (4-1bb or Tnfrsf9)* codes for two CD137 isoforms: membrane-bound (mCD137) and soluble (sCD137) forms (**Figure 2B**) (85). Membrane mCD137 is mostly found on CD4^+^ and CD8^+^ T cells, whereas the sCD137 is produced by Tregs (86). The ligand for both isoforms, CD137L, is coded by *Tnfsf9* on chromosome 17 and is expressed on APCs and activated T cells (86).

Since the NOD *Cd137* SNPs suggested that mCD137 was hypofunctional compared to the NOD.B10 strain (82), Cannons et al. (84) evaluated T cells activation and proliferation to test this hypothesis. They confirmed that NOD and NOD.B10 mice showed similar mCD137 expression after stimulation with anti-CD3 in Th1 and Th2 culture conditions. However, when T cells from NOD mice were costimulated with CD137L, they proliferated less and produced a reduced level of IL-2 than T cells from mice carrying the B10 allele of *Cd137*. This strongly suggested that the NOD SNPs lead to a hypo-functional mCD137 protein, which could play a role in T1D pathogenesis. Over the last 15 years, our work has begun to delineate the complex immune biology of CD137 and CD137L in T1D.

## CD137 and CD137L: A double-edged sword in autoimmunity

CD137 is a glycoprotein belonging to the TNF receptor superfamily, and the membrane form is expressed on activated CD4^+^ and CD8^+^ T lymphocytes (87), Tregs (88–94), and natural killer (NK) cells (95). CD137 is also constitutively expressed on a subset of Tregs (93). CD137L (4-1BBL), its ligand, belongs to the TNF superfamily and is expressed on activated APCs such as macrophages, B cells, and dendritic cells (96–98). Activated T cells also upregulate and express CD137L (99). mCD137 has no intrinsic enzymatic activity in its intracellular domain and functions by binding TRAF1 and TRAF2 adaptor proteins that enhance K63 polyubiquitination processes in the CD137 signalosome (100, 101). CD137L trimerization, in response to interaction with mCD137, causes mCD137 receptor clustering and TRAF-mediated activation of the ERK, JNK, p38, NF-kB, and MAPK intracellular signaling pathways, resulting in cell activation, proliferation, and T cell survival (102–109). Notably, signaling in the CD137:CD137L pathway is bidirectional: both the receptor and ligand signal into their respective cells (110, 111). This bidirectional signaling adds an additional layer of complexity to the analysis of the biological function of the pathway.

The effects of CD137 in T cell biology are diverse but with specific implications for inflammation and immune regulation. TCR-induced proliferation and cytokine production were enhanced after T cells were stimulated with agonistic anti-CD137 antibodies (also known as 3H3), independent of B7-CD28/CTLA-4 interactions (112, 113). mCD137 signaling results in NF-kB activation that promotes the expression of antiapoptotic genes encoding Bcl-xL and Bfl-1 (114, 115) and mitochondrial function and biogenesis, which improves T cell survival (116, 117).

mCD137 has a prominent role in CD8^+^ T-cell costimulation, influencing cytotoxicity in an IL-2-independent manner. Furthermore, CD8^+^ T cells produce a greater amount of IFN-γ after mCD137 activation (118). *In vivo* experiments showed that knockout mCD137/CD137L mice exhibited a reduced memory CD8^+^ T cell response to viruses (119–121) and decreased T cell survival (122). These findings pointed to a costimulatory involvement of mCD137 in long-lasting memory T-cell activation and enhancement of cytotoxicity and founded the basis for CD137-based therapies for cancer (85, 123–126). In contrast to these effects, when knockout mice were stimulated with CD3, T cells showed hyperresponsiveness, which indicated an additional immunosuppressive role of CD137 (105).

The expression of CD137L on APCs is increased at sites of inflammation *in vivo* (127, 128). Activating APCs by CD137L upregulated B7-1 and B7-2, and increased IL-6 and IL-12 secretion (127). CD137L is upregulated on activated T cells, and CD137L signaling is critical for CD8^+^ T cell survival *via* STAT3- and FAS-mediated pathways (129). CD4^+^ T cell activation can also be modulated by CD137L-expressing APCs (*via* APC CD137L signaling through T cell CD137) that stimulate IL-2 and IL-4 T cell production (112, 113).

This data established that CD137L on APCs affects the cytotoxic immune response and is critical for the survival of CD8^+^ and CD4^+^ T cells. This also confirmed that inhibition of mCD137 or CD137L might reduce inflammation *via* CD8^+^ T cells but may at the same time also affect CD4^+^CD25^+^CD137-expressing Tregs. Indeed, *Cd137* is upregulated by *Foxp3* (130). CD137 is expressed by Tregs infiltrating the islets in T1D, suggesting an immunoregulatory role for CD137^+^ Tregs (131). Thus attempting to modulate CD137 or CD137L action on T effector cells could potentially decrease immunosuppression *via* Tregs, illustrating the intricacies of this pathway and the potential for double-edged effects.

Type 1 regulatory T (Tr1) cells are another type of regulatory T cell characterized by the production of IL-10 and lack of constitutive *Foxp3* expression (132). Despite the evidence of CD137L mRNA expression after stimulation (133), it is unknown whether these cells also exert their suppressive function by sCD137 or their role in NOD mice during T1D pathogenesis. Since *Cd137* is upregulated by *Foxp3* (130), Tr1 cells may not produce large quantities of sCD137. Further studies of this cell subset and their involvement in the mCD137/CD137L axis are warranted.

Agonistic anti-CD137 antibodies induced the proliferation of CD4^+^CD25^+^ Tregs with the maintenance of their suppressive activity (92). Interestingly, the effects of agonistic anti-CD137 antibodies are diverse and dependent on the target and the disease. Activating CD8^+^ T cells by anti-CD137 antibodies in cancer models leads to tumor cell elimination. In sharp contrast, in models of autoimmunity, e.g., murine models of SLE (134, 135), experimental autoimmune encephalomyelitis (136), collagen-induced arthritis (122, 137), Sjögren’s syndrome-like sialadenitis (138), and inflammatory bowel disease (139), anti-CD137 antibody treatment leads to immunoregulation and disease amelioration. For example, anti-CD137 administration in the SLE murine model reversed disease and reduced autoantibody production (i.e., dsDNA antibodies) and immune complex deposition (135). Induction of T cell anergy by anti-CD137 antibodies might play a role in some of these models (135, 140).

## Anti-CD137 antibodies prevented T1D *via* Treg expansion but accelerated T1D in the absence of Tregs

Since *Cd137* was a candidate gene in T1D, we started our investigation of the role of CD137 in T1D with agonistic anti-CD137 antibodies. We showed that anti-CD137 antibodies in NOD mice prevented the development of T1D but did not ameliorate insulitis, which is consistent with the findings of residual insulitis in NOD congenic mice protected from T1D by the B10 *Idd9.3* region (93). We found that anti-CD137 expanded CD4^+^CD25^+^ Tregs, and their transfer to NOD-*scid* mice completely prevented T1D (93). However, T1D progressed more rapidly when NOD-*scid* mice were treated with anti-CD137 after pathogenic CD4^+^ and CD8^+^ T cell transfer in the absence of Tregs. Therefore, in the absence of Tregs, mCD137 stimulation could potentially potentiate pancreatic destruction *via* CD8^+^ T cytotoxicity. This is similar to the effect of anti-CD137 administered in the context of autoimmune thyroiditis, which worsens the disease (141).

Due to this dual effect, activation of effector T cells in acute autoimmunity may prohibit the use of agonistic CD137 antibodies in clinical autoinflammatory states, including T1D, because activated T cells have upregulated mCD137 in these settings. In contrast, CD137 antibodies in non-inflammatory states (e.g., pre-diabetes) might prevent autoimmunity since it targets Tregs constitutively expressing mCD137 without activating T cell effector cells. This dual effect led us to look for alternate ways to therapeutically target the mCD137/CD137L pathway in T1D.

We turned our attention to sCD137, which is formed by alternative splicing (99, 142) (**Figure 2B**), and exists as a dimer (143). sCD137 was found in the supernatants of splenic and bone marrow-derived dendritic cells (144). Murine sCD137 differs from humans. In mice, only the exon coding for the transmembrane domain of CD137 is spliced out, whereas, in the latter, two splice variants are observed (145). sCD137 is preferentially secreted by CD4^+^ T cells, whereas CD8^+^ T cells express higher amounts of mCD137 (146). We found that the major source of sCD137 is CD4^+^CD25^+^CD137^+^ Tregs (94).

Spliced variants are critical for the modulation of immune response (147). The induction of alternative splicing is poorly understood but may occur as a response to environmental signals. In autoimmunity, splicing also occurs in the modulation of immune responses (147). Changes in the immunological environment (i.e., T cell autoreactivity and pro-inflammatory milieu) induce the production of sCD137 by Tregs. We have demonstrated that activating Treg cells increases the production of sCD137 by Tregs in mice and humans (148). Thus, inflammatory environmental changes may partly explain the origin of spliced variants of CD137 from Tregs as a homeostatic response to ameliorate inflammation. A similar process is seen with soluble CTLA4 (sCTLA4), a spliced variant of membrane-bound CTLA4 mainly produced by Foxp3^+^ Tregs (149). sCTLA4 suppresses early T-cell activation by preventing the interaction of CD80/CD86 with the costimulatory receptor CD28 (150). In addition, it inhibits IFN-α, IL-2, IL-7, and IL-13 production while activating TGF-β and IL-10 release (151). Silencing sCTLA-4 mRNA by RNA interference accelerated the onset of T1D in NOD mice and impaired the ability of Tregs to downregulate dendritic cell costimulation (149). Both spliced variants, sCTLA4 and sCD137, may be critical for effective Treg function in the pathogenesis of T1D.

What is the role of sCD137? Our hypothesis was that sCD137, similar to sCTLA4, functions as a negative feedback mechanism to downregulate immune response mediated by mCD137 and CD137L (85, 152). sCD137 reduces the production of IL-10 and IL-12 from activated splenocytes (146). In addition, T cell proliferation and IL-2 release were inhibited when sCD137 was administered to these cells (153). These initial reports clarified the effects of CD137 in different conditions (i.e., cancer and autoimmunity) and suggested that the sCD137 was the missing link in understanding the dual effects of the mCD137/CD137L axis.

To confirm the role of CD137 in the *Idd9.3* locus, we evaluated the function (immunosuppressive effects) and quantity of CD137^+^ Treg cells in NOD.*Idd9.3* congenic mice (94). When compared to NOD mice, the NOD.*Idd9.3* strain had significantly higher percentages of CD4^+^CD25^+^CD137^+^Foxp3^+^ Tregs in the thymus and spleen, and the numbers increased with age. This supported the hypothesis that the hypofunctional NOD CD137 allele led to decreased Treg survival, consistent with the known effects of mCD137 on cell survival. CD137^+^ Tregs showed superior immunosuppression compared to CD4^+^CD25^+^CD137^-^ Tregs, directly showing an effect of CD137 on Treg function. Thus, increased numbers of CD137 Tregs, mediated by the protective allele, led to increased overall suppressive capacity. Importantly, CD137^+^ Tregs showed suppressive capability in an independent contact assay. This supported our continued focus on the possible immunosuppressive role of sCD137 in T1D.

## sCD137 is produced by Tregs and inhibits T cell autoreactivity in a paracrine fashion

We first confirmed that sCD137 was mainly produced by CD4^+^CD25^+^CD137^+^ Tregs and in a higher amount in NOD.*Idd9.3* congenic mice (94). Next, we demonstrated that sCD137 primarily exists as a ~55 kDa homodimer under non-reducing and a ~35 kDa monomer under reducing conditions (143). The existence of sCD137 as a dimer, rather than as a trimer as described for mCD137, suggested a structural reason for how sCD137 might suppress T cell function while mCD137 activated T cell function (143). Next, we showed that the administration of recombinant sCD137 to NOD mice prevented diabetes and reduced insulitis by preserving insulin^+^ islets (143). Since CD4^+^CD25^+^CD137^+^ Tregs inhibited T cells in a contact-independent manner (94), we evaluated the role of sCD137 in T cell inhibition. We demonstrated that sCD137 inhibited activated T cells by binding to CD137L (143). In addition, sCD137 can directly stop the proliferation of effector CD4^+^CD25^-^CD137^-^ T cells in the absence of APCs, and without inducing cell death (143) (**Figure 2C**).

In addition to the crucial role of sCD137 in immunosuppression, additional reports suggest that mCD137, like other costimulatory molecules, has a nonredundant role in maintaining the pathogenic activity of β cell-autoreactive T cells in NOD mice. We found that, compared to wild-type mice, T1D development is reduced in NOD.*Cd137* ^-^/^-^ and their T cells are less capable of inducing T1D in NOD.*Rag1^-^/^-^* recipients (154). This, at first, seemed contradictory to our data on the immunoregulatory properties of CD137^+^ Tregs and sCD137. As sCD137 produced by Tregs is suppressive, evaluating the distinctive role of mCD137 in CD4^+^ and CD8^+^ T cells was crucial. Isolated T cells from NOD and NOD.*Cd137* ^-^/^-^ mice were transferred into NOD.*Rag1^-^/^-^* recipients. The T cell adoptive transfer studies revealed that CD137 expression in CD8^+^ T cells was required to develop T1D in NOD mice, but CD137 expression in CD4^+^ T cells was diabetes-protective (155). Specifically, CD137 expression in CD4^+^ Tregs is important for their T1D suppression function. We further demonstrated that CD137 cell-intrinsically stimulates the accumulation and proliferation of autoreactive CD8^+^ T lymphocytes within the islets, pointing to a role of mCD137 on the diabetogenic activity of CD8^+^ T cells. However, sCD137 suppressed the proliferation of CD8^+^ T cells. These experiments supported the concept that the T1D protection conferred by the *Idd9.3* locus is mediated through the production of sCD137 by Tregs.

As the sCD137/CD137L interaction is implicated in the modulation of effector CD8^+^ T cells, clarifying the role of CD137L in the immunomodulation of T1D is essential. CD137L-deficient NOD mice were shown to exhibit less insulitis and delayed onset of T1D (156). Interestingly, CD137L expression on myeloid APCs appeared to be necessary for the survival of β-cell–autoreactive CD8^+^ T cells and T1D progression, but CD137L has no effect on the formation or homeostasis of Foxp3^+^ Tregs (156). It remains to be determined if mCD137 in Tregs modulates their function and whether Tregs capable of producing sCD137 but not mCD137 are sufficient to suppress T1D.

## sCD137 induces T cell anergy and can act therapeutically to halt acute autoimmunity

It is relatively easy to prevent T1D in NOD mice, and a much more stringent target is the reversal of actual acute T1D. Thus we treated NOD mice with new-onset T1D with recombinant sCD137 (148). This experiment confirmed that sCD137 could not only prevent T1D but also halt acute T1D and avert the development of end-stage diabetes. In effectively treated mice, β cell immunohistochemistry revealed considerable preservation of insulin^+^ β cells and a rise in insulin^+^ islets (148). In this setting, T cells showed downregulation of mTORC1, developed an anergic phenotype (reversed by IL-2), as well as the ability of sCD137 to suppress antigen-experienced and activated memory T cells. CD8^+^ effector memory cells also showed a reduction in the production of inflammatory cytokines in the presence of sCD137 (i.e., IFN-γ) (148).

In human pediatric T1D patients, we found low levels of sCD137 compared to non-diabetic age-matched controls during acute flares (hospital admission for hyperglycemia). We also confirmed that human Tregs were the primary source of sCD137 (148). Furthermore, human peripheral activated CD4^+^ T cells were inhibited by sCD137. These results were analogous to those in NOD congenic strains, supporting the notion that these murine models are useful and relevant to affecting the autoimmune phenomena driving human T1D. This evidence showed that sCD137 is associated with autoimmunity in T1D humans, and low sCD137 could be a biomarker in T1D. Further studies are required to confirm the role of sCD137 in reverting established destructive insulitis and the pathways associated with this phenomenon.

Surprisingly, sCD137 is reported to be increased in patients with rheumatoid arthritis (145, 157, 158), and multiple sclerosis (159); and the levels were directly correlated with the severity of the disease (157). Increased levels of sCD137 could be a homeostatic attempt by Tregs to modulate inflammation in these conditions. However, it also raises the possibility that in the presence of a substantial inflammatory substrate, the stoichiometric ratio of sCD137 to CD137L could be reduced, thus reducing the efficacy of sCD137 or possibly indicating that higher sCD137 doses would be required. New strategies improving the half-life and potency of sCD137 could be critical to enhancing their therapeutic effect in human autoimmunity.

## Summary and prospects

Linkage studies and the construction of congenic mice allowed the identification of candidate genes with implications for the pathogenesis of T1D. The cumulative evidence suggests that *Cd137* and its coding isoforms are crucial in the development of T1D, and the CD137-CD137L pathway is a good target for therapeutic modulation. Treg-generated sCD137 modulates the mCD137/CD137L axis, reduces insulitis, and halts T1D in the NOD mouse. The ability to effectively halt acute T1D with exogenous sCD137 is an exciting development with attractive therapeutic potential. Prevention studies in humans are difficult to implement, and those attempted so far have failed (160). Therefore, treating acute disease is a more appealing strategy, but the current landscape of approved therapeutics is limited. The use of antibodies to target the CD137-CD137L axis is appealing; however, while anti-CD137 antibodies are protective in some models of autoimmune diseases due to activation of Tregs, they can also enhance CD8^+^ T cell killing activity in the absence of Tregs. sCD137, on the other hand, only acts to suppress CD4^+^ and CD8^+^ T cell activation and may therefore be safer than an anti-CD137 based approach. In human studies, low levels of sCD137 during T1D flares, and the inhibition of activated CD8^+^ T cells *in vitro* after sCD137 stimulation, supports its further translational use. Soluble CD137 suppresses autoreactive CD8^+^ T cells through induction of anergy. However, little is known about the activity of sCD137 on innate immunity. The mechanistic role of sCD137 on CD137L-expressing myeloid APCs should be explored to determine if there will be lasting effects on innate immune function. In addition, it is unknown whether mCD137 on Tregs drives their differentiation to a more robust inhibitory phenotype. This could have therapeutic implications, particularly for the pharmacokinetics and pharmacodynamics of human sCD137.

## Author contributions

MR, LH, WZ, YG, WR wrote, reviewed and revised the manuscript. All authors contributed to the article and approved the submitted version.

## Funding

This work was supported by R01 DK107541 (Y-GC and WMR) and DK121747 (Y-GC).

## Conflict of interest

The authors declare that the research was conducted in the absence of any commercial or financial relationships that could be construed as a potential conflict of interest.

## Publisher’s note

All claims expressed in this article are solely those of the authors and do not necessarily represent those of their affiliated organizations, or those of the publisher, the editors and the reviewers. Any product that may be evaluated in this article, or claim that may be made by its manufacturer, is not guaranteed or endorsed by the publisher.

## Abbreviations

ADs, Autoimmune diseases; APCs, Antigen-presenting cells; CD, Cluster of differentiation; CD137L, CD137 Ligand; *Foxp3*, Forkhead box P3; *Idd*, Insulin-dependent diabetes loci; IPEX, Immune dysregulation polyendocrinopathy enteropathy X-linked; mCD137, Membrane-bound CD137; NK, Natural killer; NOD, Non-obese diabetic; NON, Non-obese non-diabetic; sCD137, Soluble CD137; sCTLA4, Soluble CTLA4; SLE, Systemic lupus erythematosus; SNPs, Single nucleotide polymorphisms; T1D, Type 1 diabetes; Tregs, T regulatory cells.
